# Downregulation of *Ripk1* and *Nsf* mediated by CRISPR-CasRx ameliorates stroke volume and neurological deficits after ischemia stroke in mice

**DOI:** 10.3389/fnagi.2024.1401038

**Published:** 2024-06-11

**Authors:** Xincheng Song, Yang Lan, Shuang Lv, Yuye Wang, Leian Chen, Tao Lu, Fei Liu, Dantao Peng

**Affiliations:** ^1^Peking University China-Japan Friendship School of Clinical Medicine, Beijing, China; ^2^Department of Neurology, China-Japan Friendship Hospital, Beijing, China; ^3^Department of Cardiovascular Medicine, The Second Affiliated Hospital of Nanchang University, Nanchang, China; ^4^Department of Rheumatology, The First Hospital of Hebei Medical University, Shijiazhuang, China; ^5^China-Japan Friendship Hospital (Institute of Clinical Medical Sciences), Chinese Academy of Medical Sciences & Peking Union Medical College, Beijing, China; ^6^Institute of Neuroscience, State Key Laboratory of Neuroscience, CAS Center for Excellence in Brain Science and Intelligence Technology, Shanghai Research Center for Brain Science and Brain-Inspired Intelligence, Shanghai Institutes for Biological Sciences, Chinese Academy of Sciences, Shanghai, China; ^7^Anhui Province Key Laboratory of Clinical and Preclinical Research in Respiratory Disease, Molecular Diagnosis Center, Department of Pulmonary and Critical Care Medicine, First Affiliated Hospital, Bengbu Medical College, Bengbu, China

**Keywords:** CRISPR-CasRx, ischemic stroke, ripk1, NSF, AAV

## Abstract

Necroptosis is implicated in the pathogenesis of ischemic stroke. However, the mechanism underlying the sequential recruitment of receptor-interacting protein kinase 1 (RIPK1) and N-ethylmaleimide-sensitive fusion ATPase (NSF) in initiating necroptosis remains poorly understood, and the role of NSF in ischemic stroke is a subject of controversy. Here, we utilized a recently emerging RNA-targeting CRISPR system known as CasRx, delivered by AAVs, to knockdown *Ripk1* mRNA and *Nsf* mRNA around the ischemic brain tissue. This approach resulted in a reduction in infarct and edema volume, as well as an improvement in neurological deficits assessed by Bederson score, RotaRod test, and Adhesive removal test, which were achieved by RIPK1/receptor-interacting protein kinase 3/mixed lineage kinase domain-like protein signaling pathway involved in neuronal necroptosis. In conclusion, the downregulation of *Ripk1* mRNA and *Nsf* mRNA mediated by CRISPR-CasRx holds promise for future therapeutic applications aimed at ameliorating cerebral lesions and neurological deficits following the ischemic stroke.

## Introduction

Ischemic stroke is a predominant contributor to global mortality and disability (Tsao et al., 2023). Necroptosis, a distinct nonapoptotic cell death pathway characterized by necrotic cellular morphology and activation of autophagy, has been implicated in the pathogenesis of ischemic stroke (Degterev et al., 2005). Determining whether the molecules implicated in necroptosis can serve as potential targets holds the key to developing future therapies for patients with ischemic stroke (Deng et al., 2019; Zhang et al., 2020; Li et al., 2023).

Recent studies have revealed the critical involvement of N-ethylmaleimide-sensitive fusion ATPase (NSF) in disrupting the auto-inhibition effect of acid-sensing ion channel 1a (ASIC1a), enabling the recruitment of receptor-interacting protein kinase 1 (RIPK1) to interact with the N-terminus of ASIC1a. This interaction triggers neuronal death under acidosis induced by ischemic stroke (Wang et al., 2020). However, the precise mechanism by which NSF and RIPK1 cooperate to initiate necroptosis remains unclear, and the role of NSF in ischemic stroke is a subject of controversy (Matsushita et al., 2005; Zou et al., 2005; Yuan et al., 2018a). Furthermore, the *in vivo* downregulation of *Ripk1* and *Nsf* has not been thoroughly explored. An emerging characterized RNA-guided and RNA-targeting CRISPR protein family, known as Cas13, offers a novel approach to manipulate RNA transcripts (Abudayyeh et al., 2017; Cox et al., 2017; Knott and Doudna, 2018). Among the Cas13 proteins, the ortholog of CRISPR-Cas13d (CasRx) stands out because its compact size, exceptional targeting specificity, and efficiency, making it well-suited for *in vivo* therapeutic applications (Konermann et al., 2018).

In this study, we demonstrated that the specific knockdown of *Ripk1* mRNA and *Nsf* mRNA in the striatum and primary somatosensory cortex-barrel field (S1BF) through local injection of adeno-associated viruses (AAVs) expressing CasRx and two guide RNAs (gRNAs) targeting *Ripk1* mRNA and *Nsf* mRNA resulted in a reduction of infarct and edema volume, as well as an improvement in neurological deficits in a mouse model of transient middle cerebral artery occlusion (tMCAO). The simultaneous knockdown of *Ripk1* and *Nsf* exerts its effects on ischemic stroke through the RIPK1/RIPK3/ mixed lineage kinase domain-like protein (MLKL) signaling pathway involved in necroptosis. Therefore, the CasRx-mediated knockdown of *Ripk1* and *Nsf* holds promise for future therapeutic applications aimed at ameliorating cerebral lesions and neurological deficits following ischemic stroke.

## Materials and methods

### Animals

Adult male C57BL/6 J mice were purchased from Shanghai SLAC Laboratory Animal Co., Ltd. and raised to 12 weeks-old (25 to 30 g) for experiments. All mice used in this study were housed in a 12 h light/dark cycle room with water and food *ad libitum*. All animal procedures were performed in compliance with the guidelines and regulations approved by the Institutional Animal Care and Use Committee of Fudan University (ethical approval number: B2022-031R).

### Cell line

The Neuro-2a (N2a) cell line, obtained from the Cell Bank of Shanghai Institute of Biochemistry and Cell Biology (SIBCB), Chinese Academy of Sciences (CAS), was cultured in DMEM supplemented with 10% FBS and 1% penicillin/streptomycin. The cells were maintained in a 37°C incubator with 5% CO_2_.

### Transfection, qPCR, and RNA-seq

Transient transfection was carried out using 4 μg vectors expressing U6-gRNA-EF1α-CasRx-eGFP according to the standard procedure with Lipofectamine 3,000 (Thermo Fisher Scientific). For the screening of *Ripk1* gRNAs (Supplementary Table S1), the experimental plasmids included U6-*Ripk1* (gRNA)-EF1α-CasRx-eGFP along with CAG-*Ripk1*-mCherry, while the control plasmid consisted of CAG-*Ripk1*-mCherry and U6-EF1α-CasRx-eGFP (no gRNA). Similarly, for the screening of *Nsf* gRNAs (Supplementary Table S1), the experimental plasmid was U6-*Nsf* (gRNA)-EF1α-CasRx-eGFP, and the control plasmid was U6-EF1α-CasRx-eGFP (no gRNA). N2a cells were seeded in 6-well plates and transfected with 4 mg vectors expressing U6-gRNA-EF1α-CasRx-eGFP using Lipofectamine 3,000. Two days post-transfection, approximately 50,000 cells per sample, positive for both eGFP and mCherry (for *Ripk1* gRNAs) or positive for eGFP (for *Nsf* gRNAs), were collected by fluorescence activating cell sorting (FACS, MoFlo XDP, Beckman Coulter Ltd.) and lysed for qPCR analysis. RNA was extracted using Trizol (Ambion), and cDNA was synthesized using a reverse transcription kit (HiScript Q RT SuperMix for qPCR, Vazyme, Biotech). The qPCR Primers of *Ripk1* and *Nsf* were shown in Supplementary Table S2. For RNA-seq, N2a cells were cultured in a 15 cm dish, and transient transfection was performed with 70 μg plasmids. The experimental plasmids included U6-*Ripk1* (gRNA)-*Nsf* (gRNA)-EF1α-CasRx-eGFP and CAG-*Ripk1*-mCherry, and the control plasmid consisted of CAG-*Ripk1*-mCherry and U6-EF1α-CasRx-eGFP (no gRNA). Approximately 300,000 eGFP-positive and mCherry-positive (top 20%) N2a cells were collected by FACS. Subsequently, RNA was extracted and then converted to cDNA, which was used for transcriptome-wide RNA-seq analysis. High-throughput mRNA sequencing was performed using the Illumina Genome Analyzer, and adapter removal was carried out using Trimmomatic (v0.36) during the sequencing process. The hisat2 (v2.0.0) tool was employed to map qualified reads to the mouse reference genome (mm10) with default parameters. The expression levels of all mapped genes were estimated using stringtie (v2.0), and the gene expression abundances were indicated by FPKM (fragments per kilobase of transcript per million fragments mapped).

### AAV preparation

Due to the packaging capacity limitations of AAV, we reconstructed U6-*Ripk1* (gRNA)-*Nsf* (gRNA)-EF1α-CasRx-eGFP by omitting eGFP. A gRNA for *Ripk1* and a gRNA for *Nsf* were cloned into an adeno-associated viral backbone vector, where two gRNAs were driven by a U6 promoter and CasRx was driven by a EF1α promoter, forming the target plasmid EF1α-CasRx-*Ripk1*-*Nsf*. And the adeno-associated viral backbone vector itself can serve as a control for the target plasmid, which named as EF1α-CasRx. The adeno-associated viral backbone vector and the plasmid of EF1α-mCherry were a gift from Canbing Feng. The plasmids EF1α-CasRx-*Ripk1*-*Nsf*, EF1α-CasRx and EF1α-mCherry were custom-packaged using AAV9 by OBiO Technology (Shanghai) Corp., Ltd.

### Stereotactic injection

The mice were anesthetized using 3% isoflurane for induction and 1.5% for maintenance. Subsequently, they were secured in a stereotactic frame. AAVs containing either AAV-EF1α-CasRx-*Ripk1*-*Nsf* plus AAV-EF1α-mCherry or AAV-EF1α-CasRx plus AAV-EF1α-mCherry was injected into striatum (AP = 0 mm, ML = +2.2 mm, DV = −2.8 mm) and S1BF (AP = −0.5 mm, ML = +3.2 mm, DV = −1.3 mm) using a micromanipulator at a slow rate (approximately 6 min per microliter). Mice were injected in the striatum and S1BF with high-titer AAVs (>1 × 10^13^ vg/mL, 1 μL per site). The volume ratio between AAV-EF1α-mCherry and AAV-EF1α-CasRx-*Ripk1*-*Nsf* or AAV-EF1α-CasRx was 1:9.

### Transient middle cerebral artery occlusion

The mice were anaesthetized using 3% isoflurane for induction and 1.5% for maintenance. Body temperature was carefully maintained at 37°C using a thermostatically controlled heating pad. Meloxicam (6 mg/kg) was administered subcutaneously 30 min before surgery for analgesia. A midline cervical incision was made, and under an operating microscope, the left common carotid artery (CCA), external artery (ECA), and internal carotid artery (ICA) were exposed. A 6-0 silicone-coated monofilament (602356PK5Re, Doccol Ltd.) was inserted into the CCA and advanced into the ICA approximately 9–11 mm from the common carotid bifurcation. The monofilament was kept in place for 60 min. After removing the monofilament and providing wound care, the animals were returned to their cages and closely monitored for 24 h. The same procedure was performed for sham-operated animals, except that the advancement distance of the monofilament was reduced to 5 mm from the common carotid bifurcation.

### 2,3,5-Triphenyltetrazolium chloride staining and stroke volume

Coronal slices (1 mm thick) of murine brain were immersed in a 2% solution of TTC (298-96-4, Sangon Biotech Ltd., Shanghai, China) at 37°C for 10 min. At the 5th minute, the slices were flipped over. The stroke areas were quantified using ImageJ software. To compensate for cerebral swelling (oedema) and overestimation of the infarct volume, we used the following formula: percentage of corrected infarct volume = (contralateral hemisphere volume − (ipsilateral hemisphere volume − infarct volume))/total brain volume. Similarly, the percentage of corrected oedema volume = (ipsilateral hemisphere volume − contralateral hemisphere volume)/total brain volume.

### Western blot

Snap-frozen brain tissue was homogenized with ice-cold RIPA lysis buffer (P0013K, Beyotime Ltd.), and the supernatant was collected. 30 μg of total protein lysate was used for sodium dodecyl sulfate polyacrylamide gel electrophoresis (SDS-PAGE) and transferred onto polyvinylidene fluoride (PVDF) membranes (IPVH00010, Merk Millipore). The membranes were incubated overnight at 4°C with the following primary antibodies: rabbit monoclonal anti-RIPK1 (1:1000, 3,493, Cell Signaling Technology), rabbit monoclonal anti-RIPK3 (1:1000, 10,188, Cell Signaling Technology), rabbit monoclonal anti-MLKL (1:1000, 37,705, Cell Signaling Technology), and rabbit monoclonal anti-β-actin (1:30000, AC308, Abclonal). Primary antibody dilution buffer (P0023A-500 mL, Beyotime Ltd.) and secondary antibody dilution buffer (P0023D-500 mL, Beyotime Ltd.) were used in this study. After incubation with the corresponding peroxidase-conjugated secondary antibody (anti-rabbit IgG, 1:5000, M21002, Abmart), the signal was visualized using the Omni-ECL^™^ Enhanced Pico Light Chemiluminescence Kit (Epizyme Ltd., Shanghai, China) and quantified using Image J software (V1.48, National Institutes of Health, United States). β-actin was used for signal normalization primary antibody dilution buffer (P0023A-500 mL, Beyotime Ltd.) and secondary antibody dilution buffer (P0023D-500 mL, Beyotime Ltd.)

### Co-immunoprecipitation

Snap-frozen brain tissue was homogenized with ice-cold immunoprecipitants lysis buffer (G2038-100ML, Servicebio Ltd.), and the supernatant was incubated on ice for 40 min, followed by centrifugation at 13,000 × g for 15 min. The supernatant was then incubated with 4 μg of the antibody at 4°C overnight. Protein G agarose beads (20 μL, Thermo Fisher Scientific) were added to the sample and incubated for 2 h at 4°C. The immunoprecipitants were washed three times with the lysis buffer, suspended with 2 × loading buffer, boiled, and run on SDS-PAGE. The proteins were transferred to PVDF membranes for immunoblotting. Additionally, aliquots of the original lysates were subjected to SDS-PAGE in parallel for immunoblotting to determine the input amount. The primary antibodies used were rabbit monoclonal anti-ASIC1a (1:100, ab284406, Abcam), mouse monoclonal anti-RIPK1 (1:1000, sc-133102, Santa Cruz), mouse monoclonal anti-NSF (1:1000, 515,043, Santa Cruz), and β-actin (1:5000, AC308, Abclonal). This was followed by incubation with horse radish peroxidase-conjugated secondary antibodies (Goat anti-rabbit IgG, 1:5000, GB23303; Goat anti-mouse IgG, 1:5000, GB23301; Donkey anti-goat IgG, 1:5000, GB23404, Servicebio, Shanghai, China) and visualization with enhanced chemiluminescence on an ImageQuant LAS 4000 mini digital imaging system (GE Healthcare Life Sciences). The primary and secondary antibodies were diluted in PBST with 1% BSA. The band intensities of the western blots were quantified using ImageJ with background subtraction.

### Immunofluorescence staining

The brains were perfused and fixed with 4% paraformaldehyde (PFA) overnight, followed by storage in 30% sucrose for 24 h. After embedding and freezing, the brains were sectioned with the thickness of 30 mm for immunofluorescence staining. The brain sections were thoroughly rinsed with 0.1 M phosphate-buffered saline (PBS). The following primary antibodies were used in this study: Guinea Pig anti-NeuN antibody (1:500, ABN90, Millipore), Mouse anti-Flag (1:1500, F3165, Sigma), Rabbit anti-RIPK1 (1:100, A7414, Abclonal), Rabbit anti-RIPK3 (1:100, A5431, Abclonal), Rabbit anti-MLKL (1:100, A5579, Abclonal), and Rabbit anti-NSF (1:100, A0926, Abclonal). The following secondary antibodies were used: Alexa Fluor^®^ 488 AffiniPure Donkey Anti-Rabbit IgG (H + L) (1:500, 711-545-152, Jackson ImmunoResearch), Cy5-AffiniPure Donkey Anti-Guinea Pig IgG (H + L) (1:500, 706-175-148, Jackson ImmunoResearch), and Goat anti-mouse IgG Alexa Fluor 594 (1:500, Abcam Cat# 150116). After antibody incubation, the slices were washed and covered with mountant (Life Technology). The images were visualized using an Olympus FV3000 microscope and image acquisition software is OLYMPUS FV31S-SW.

### Neurological deficits assessment

An experimenter blinded to the group allocation evaluated the neurological deficits after tMCAO. The neurological status was assessed at 2, 24, and 48 h after tMCAO using a five-point scale based on the Bederson score (Bieber et al., 2019). The scores were assigned as follows: score 0, no observable deficits; score 1, forelimb flexion; score 2, forelimb flexion and decreased resistance to lateral push; score 3, circling; score 4, circling and spinning around the cranial-caudal axis; score 5, no spontaneous movement. Neurological performance was also evaluated using the RotaRod (Cat. No. 47600, UGO BASILE S.R.L.) test and Adhesive removal test. In the RotaRod test, animals were placed on a rotating rod that gradually increased in speed (4–44 revolutions/min), and the latency to fall was recorded. The average staying time on the rod in three trials conducted one day before surgery was considered as the baseline value. Following surgery, the mean value of four trials was used as the time on the rod for the tested day. In the adhesive removal test, a 3 mm × 3 mm piece of adhesive tape was placed on the palmar surface of the contralateral forepaw. The time to contact and completely remove the tape from the forepaw was recorded, respectively. Three trials were conducted daily for each animal, starting from 3 days before surgery and continuing until specific time points after surgery. The mean value of three trials conducted one day before surgery was considered as the baseline value.

### Statistical analyses

**Figure 1:**
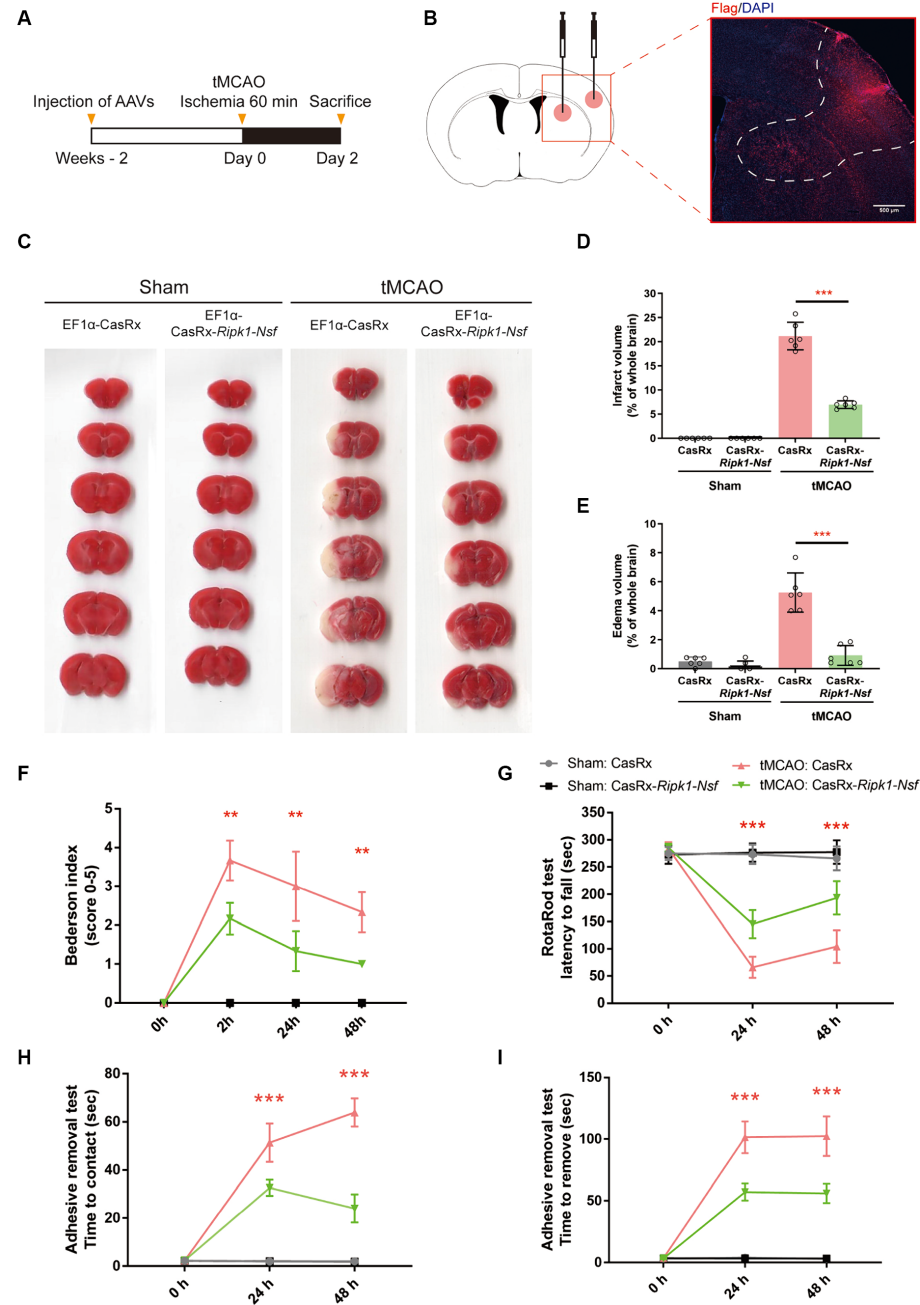
Impact of *Ripk1* and *Nsf* knockdown on cerebral lesion and neurological deficits after transient middle cerebral artery occlusion in mice. **(A)** Experimental designoverview: Four groups of mice were unilaterally injected with specific combinations of viral vectors into the left striatum and S1BF regions. The injected viral vector combinations were as follows: AAV-EF1α-CasRx-*Ripk1*-*Nsf* plus AAV-EF1α-mCherry; AAV-EF1α-CasRx plus AAV-EF1α-mCherry; AAV-EF1α-CasRx-*Ripk1*-*Nsf* plus AAV-EF1α-mCherry; AAV-EF1α-CasRx plus AAV-EF1α-mCherry. After 2 weeks period, the mice underwent a 60 min tMCAO. Subsequently, the mice were sacrificed 2 days after tMCAO. **(B)** Schematic representation of brain slice fractions illustrating the injected and infected areas of AAVs through immunofluorescence staining, specifically by examining the colocalization of Flag and DAPI. Scale bar, 500 μm. **(C–E)** Representative images of brain slices stained with 2,3,5-triphenyltetrazolium chloride and summary data indicates that a reduction in infarct volumes and edema volume in mice 48 h after tMCAO in CasRx-*Ripk1*-*Nsf* group, compared with vehicle group. *n* = 6, data are presented as mean ± SEM, *p < 0.05, **p < 0.01, ***p < 0.001, by unpaired *t* test. **(F–I)** Administration of CasRx-*Ripk1*-*Nsf* improved post-stroke neurological function in mice, as evaluated by Bederson-based neurological score, RotaRod test, and Adhesive Removal test, compared with vehicle group. *n* = 6, data are presented as mean ± SEM, *p < 0.05, **p < 0.01, and ***p < 0.001, Mann–Whitney U test for Bederson score, unpaired *t* test for RotaRod test and Adhesive Removal test.

The figures and figure legends provided information about the number of mice used in each experiment. All values were analyzed using SPSS 24.0 software (SPSS Inc., Chicago, IL, United States) and were presented as mean ± SEM, except for Figure 1F, which was presented as median (interquartile range). The statistical significance was determined using unpaired two-tailed Student’s *t* test (*p* < 0.05), except for Figure 1F, where the Mann–Whitney U test was employed. Randomization was applied to all experiments, and sample sizes were not predetermined using statistical methods. The experimenters remained blinded to group allocation during data Research Topic to avoid experimenter bias.

## Results

### Cerebral ischemia/reperfusion specifically increases the levels of RIPK1 and NSF bound to ASIC1a

**Figure 2:**
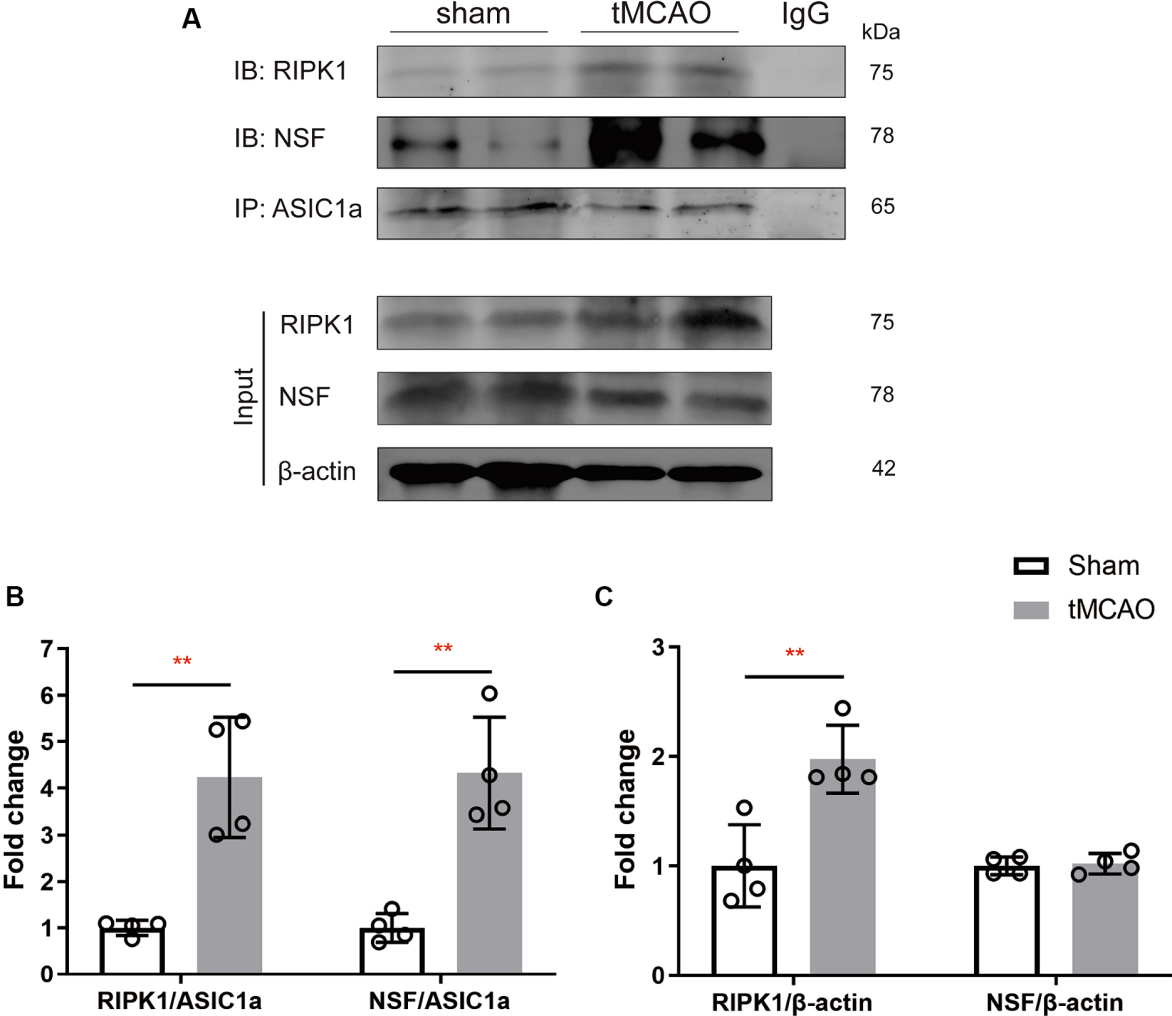
The recruitment of RIPK1 and *Nsf* to ASIC1a occurs during ischemic brain injury in mice. **(A)** The expression levels of RIPK1 and NSF, which were pulled down from ASIC1a using co-immunoprecipitation, were measured in sham-operated brain tissue and ipsilateral brain peri-infarct tissue 2 h after sham/transient middle cerebral artery occlusion. **(B,C)** The summary data indicates an increased association between RIPK1 and ASIC1a, as well as between NSF and ASIC1a, in peri-infarct brain tissue compared to sham-operated brain tissue. Additionally, the total levels of RIPK1 increased after brain ischemic injury, while NSF levels remained unchanged. *n* = 4, data are presented as mean ± SEM, *p < 0.05, **p < 0.01, ***p < 0.001, by unpaired *t* test.

To explore the specific pathophysiological relevance of RIPK1 and NSF binding to ASIC1a in the context of ischemic brain injury, we assessed the expression levels of RIPK1 and NSF in the sham-operation or the peri-infarct region of ipsilateral brain, 2 h following sham-operation/transient middle cerebral artery occlusion. Co-immunoprecipitation was carried out to isolate RIPK1 and NSF bound to ASIC1a. The result demonstrated an enhanced interaction between RIPK1 and ASIC1a (4.24 ± 1.29 vs. 1.00 ± 0.16, *p* = 0.003, Figure 2B), as well as between NSF and ASIC1a (4.33 ± 1.20 vs. 1.00 ± 0.31, *p* = 0.002, Figure 2B), in the peri-infarct brain tissue compared with the sham-operated brain tissue. Furthermore, the total levels of RIPK1 increased after brain ischemic injury (1.98 ± 0.31 vs. 1.00 ± 0.38, *p* = 0.007, Figure 2C), while NSF levels remained unchanged (1.02 ± 0.09 vs. 1.00 ± 0.08, *p* = 0.76, Figure 2C). These findings suggest an enhanced interaction of RIPK1 and NSF with ASIC1a during cerebral ischemia/reperfusion.

### Specific knockdown of *Ripk1* mRNA and *Nsf* mRNA *in vitro* using CasRx

**Figure 3:**
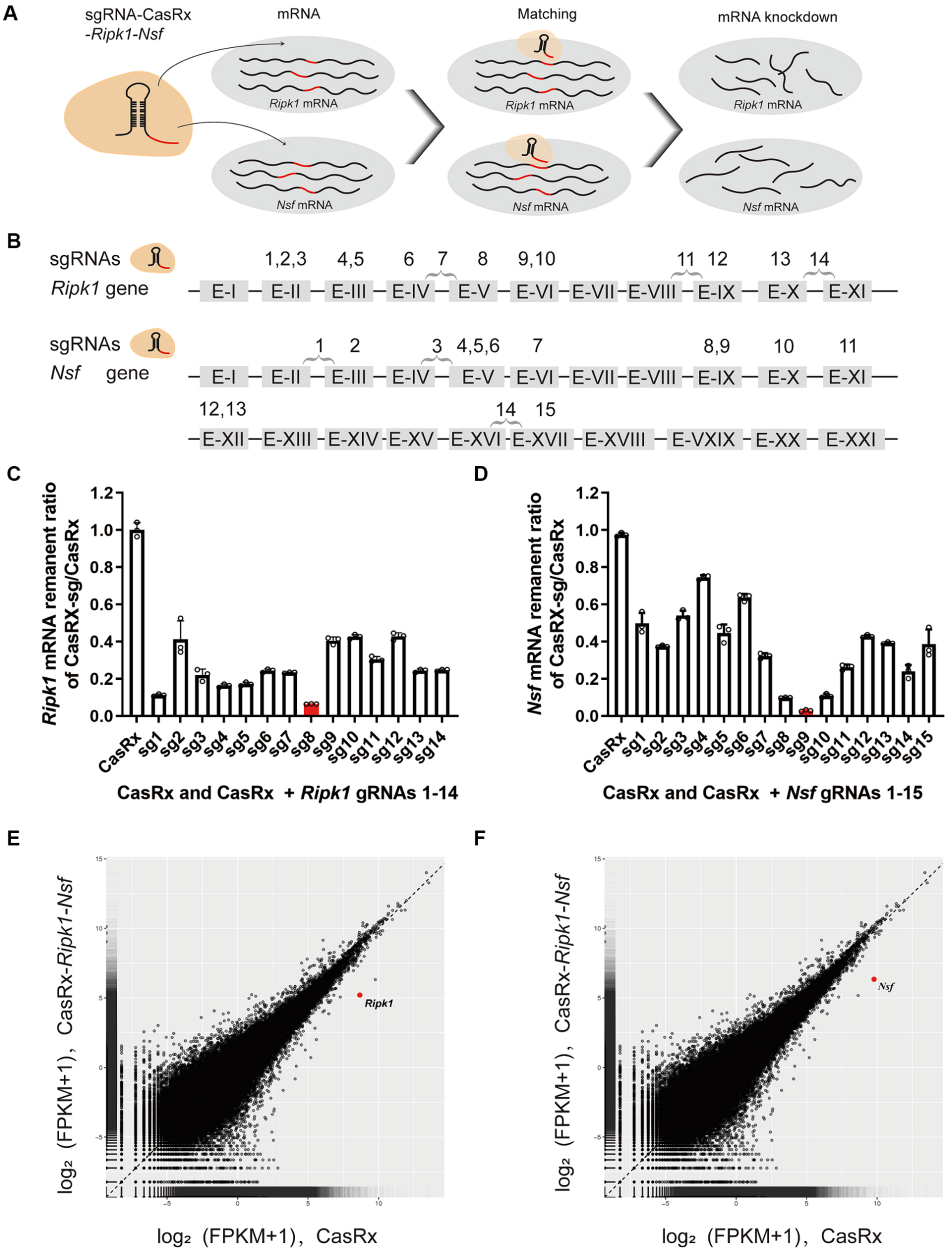
Specific Knockdown of *Ripk1* mRNA and *Nsf* mRNA *In Vitro* Using CasRx. **(A)** Schematic illustration depicting CasRx-mediated knockdown of *Ripk1* mRNA and *Nsf* mRNA. **(B)** Schematic indicating the target sites of fourteen gRNAs for *Ripk1* and fifteen gRNAs for *Nsf*. **(C,D)** The knockdown efficiency of various gRNA sites. Among them, the gRNA 8 exhibited the highest knockdown efficiency for *Ripk1*, while the gRNA 9 showed the highest knockdown efficiency for *Nsf*. *n* = 3 independent replicates for both groups. All values are presented as mean ± SEM. **(E,F)** Expression levels in log2 (fragments per kilobase per million mapped reads [FPKM] + 1) values of all detected genes in RNA sequencing (RNA-seq) libraries of CasRx-*Ripk1*-*Nsf* (*y*-axis) compared with CasRx control (*x*-axis). N2a cells, *n* = 3 independent replicates for both groups.

To evaluate the efficiency of CasRx-mediated knockdown of *Ripk1* mRNA and *Nsf* mRNA, we initially screened fourteen gRNAs targeting *Ripk1* and fifteen gRNAs targeting *Nsf* to assess their editing efficiency in N2a cells (Figures 3A,B). The gRNAs were designed to target exons, since introns are spliced out during mRNA processing. We found that the co-transfection of a vector containing the CasRx gene, a vector containing the *Ripk1* gene, and a vector containing gRNA 8 targeting *Ripk1* exon V, resulted in a significant reduction of *Ripk1* mRNA by 93.54% ± 0.09% (*n* = 3 repeats) in N2a cells, as determined by qPCR (Figure 3C). Among the gRNAs targeting *Nsf* exons, gRNA 9, which targets *Nsf* exon IX exhibited the highest knockdown efficiency, resulting in a reduction of *Nsf* mRNA by 97.12% ± 0.48% (*n* = 3 repeats) in N2a cells (Figure 3D). Consequently, gRNA 8 for *Ripk1* and the gRNA 9 for *Nsf* were co-loaded into a single plasmid along with CasRx gene, which was subsequently used in *in vivo* experiments. To evaluate the specificity of the CasRx-*Ripk1*-*Nsf* plasmid in knocking down *Ripk1* mRNA and *Nsf* mRNA, we co-transfected a vector containing the *Ripk1* gene and the CasRx-*Ripk1*-*Nsf* plasmid into N2a cells. Subsequently, we performed a transcriptome analysis to examine the expression levels of all detected genes in the RNA sequencing (RNA-seq) libraries. The analysis revealed a specific downregulation of *Ripk1* and *Nsf*, while the transcriptional levels of all other detected genes in the RNA-seq libraries remained unchanged 48 h post co-transfection (Figures 3E,F).

### Administration of AAV-EF1α-CasRx-*Ripk1*-*Nsf* reduces infarct size, edema volume, and preserves post-stroke neurological function after transient middle cerebral artery occlusion

Firstly, we examined the effect of *Ripk1* and *Nsf* knockdown *in vivo* after tMCAO. The results showed a decreased association between RIPK1 and ASIC1a (0.21 ± 0.11 vs. 1.00 ± 0.06, *p* < 0.0001, *n* = 4, Supplementary Figures S1A,B), as well as between NSF and ASIC1a (0.29 ± 0.13 vs. 1.00 ± 0.20, *p* = 0.0019, *n* = 4, Supplementary Figures S1A,B), in mice treated with CasRx-*Ripk1*-*Nsf* compared with CasRx group after tMCAO. Furthermore, the total expression levels of RIPK1 and NSF also decreased in CasRx-*Ripk1*-*Nsf* group compared with CasRx group (RIPK1: 0.29 ± 0.11 vs. 1.00 ± 0.25, *p* = 0.0019, *n* = 4, Supplementary Figures S1A,C; NSF: 0.73 ± 0.09 vs. 1.00 ± 0.11, *p* = 0.0087, *n* = 4, Supplementary Figures S1A,C). Moreover, the colocalization of RIPK1 and Flag (Supplementary Figure S1D), as well as NSF and Flag (Supplementary Figure S1E), also shown a similar tendency after tMCAO in CasRx-*Ripk1*-*Nsf* group compared with CasRx group, evaluated by immunofluorescence staining. Given the high knockdown efficiency of *Ripk1* and *Nsf in vivo* after tMCAO, we subsequently determined the therapeutic potential of *Ripk1* and *Nsf* knockdown on stroke outcome through injecting wild-type mice with AAV-EF1α-CasRx-*Ripk1*-*Nsf* (containing gRNA 8 for *Ripk1* and gRNA 9 for *Nsf*) into the S1BF and striatum 14 days prior to tMCAO/sham operation (Figure 1A), along with AAV-EF1α-mCherry, which served as a fluorescent marker indicating the infected area of the virus (Figure 1B). As a control, we used AAV- EF1α-CasRx that does not contain gRNA of *Ripk1* and *Nsf*. Stroke size, edema and neurological deficits were assessed using the Bederson score, RotaRod test, and Adhesive removal test to determine the impact of *Ripk1* and *Nsf* knockdown after tMCAO. Following 48 h of reperfusion, mice treated with AAV-EF1α-CasRx-*Ripk1*-*Nsf* exhibited a ~ 14% reduction in corrected infarct volume (21.17% ± 2.87% vs. 6.96% ± 0.81%, *p* < 0.0001, *n* = 6, Figures 1C,D), as determined by TTC staining. Moreover, knockdown of *Ripk1* and *Nsf* also decreased the corrected edema volume (5.25% ± 1.35% vs. 0.91% ± 0.68%, *p* < 0.0001, *n* = 6, Figures 1C,E). The reduction in infarct and edema volume in the CasRx-*Ripk1*-*Nsf* group following tMCAO suggests that the knockdown of *Ripk1* and *Nsf* provides a certain level of protection against ischemia brain injury. We observed a tendency towards improved neurological performance, as evaluated by the Bederson score, in the CasRx-*Ripk1*-*Nsf* group at 2 h, 24 h, and 48 h after tMCAO, compared with the vehicle-treated group (data presented as median [interquartile range]; 2 h: 4.00 (1.00) vs. 2.00 (0.25), *p* = 0.004; 24 h: 3.00 (2.00) vs. 1.00 (1.00), *p* = 0.009; 48 h: 2.00 (1.00) vs. 1.00 (0), *p* = 0.002; *n* = 6, Figure 1F). Additionally, the results of RotaRod test demonstrated a significantly prolonged latency to fall from the rotating rod in the CasRx-*Ripk1*-*Nsf* group at 24 h and 48 h after tMCAO, compared with the vehicle group (24 h: 65.88 ± 19.34 vs. 145.38 ± 25.68 s, *p* < 0.0001; 48 h: 104.04 ± 30.25 vs. 193.50 ± 30.55 s, *p* < 0.0001, *n* = 6, Figure 1G). Similarly, mice in CasRx-*Ripk1*-*Nsf* group exhibited faster response times to touch and to remove the adhesive tape at 24 h and 48 h after tMCAO, compared with the vehicle group(time to touch at 24 h: 51.39 ± 7.94 vs. 32.67 ± 3.39 s, *p* < 0.0001; time to touch at 48 h: 63.89 ± 5.87 vs. 24.06 ± 5.83 s, *p* < 0.0001, *n* = 6, Figure 1H; time to remove at 24 h: 101.5 ± 12.90 vs. 57.07 ± 6.86 s, *p* < 0.0001; time to remove at 48 h: 102.45 ± 16.01 vs. 56.00 ± 7.79 s, *p* < 0.0001, *n* = 6, Figure 1I). These findings suggest that mice in the CasRx-*Ripk1*-*Nsf* group exhibited significantly less sensorimotor deficits and better neurological recovery than vehicle-treated group. Overall, these results indicate that knockdown of *Ripk1* and *Nsf* improves histological outcome and neurological recovery after tMCAO in mice.

### Knockdown of *Ripk1* and *Nsf* mediated by CasRx ameliorate necroptosis in neurons through RIPK1/RIPK3/MLKL signaling pathway

**Figure 4:**
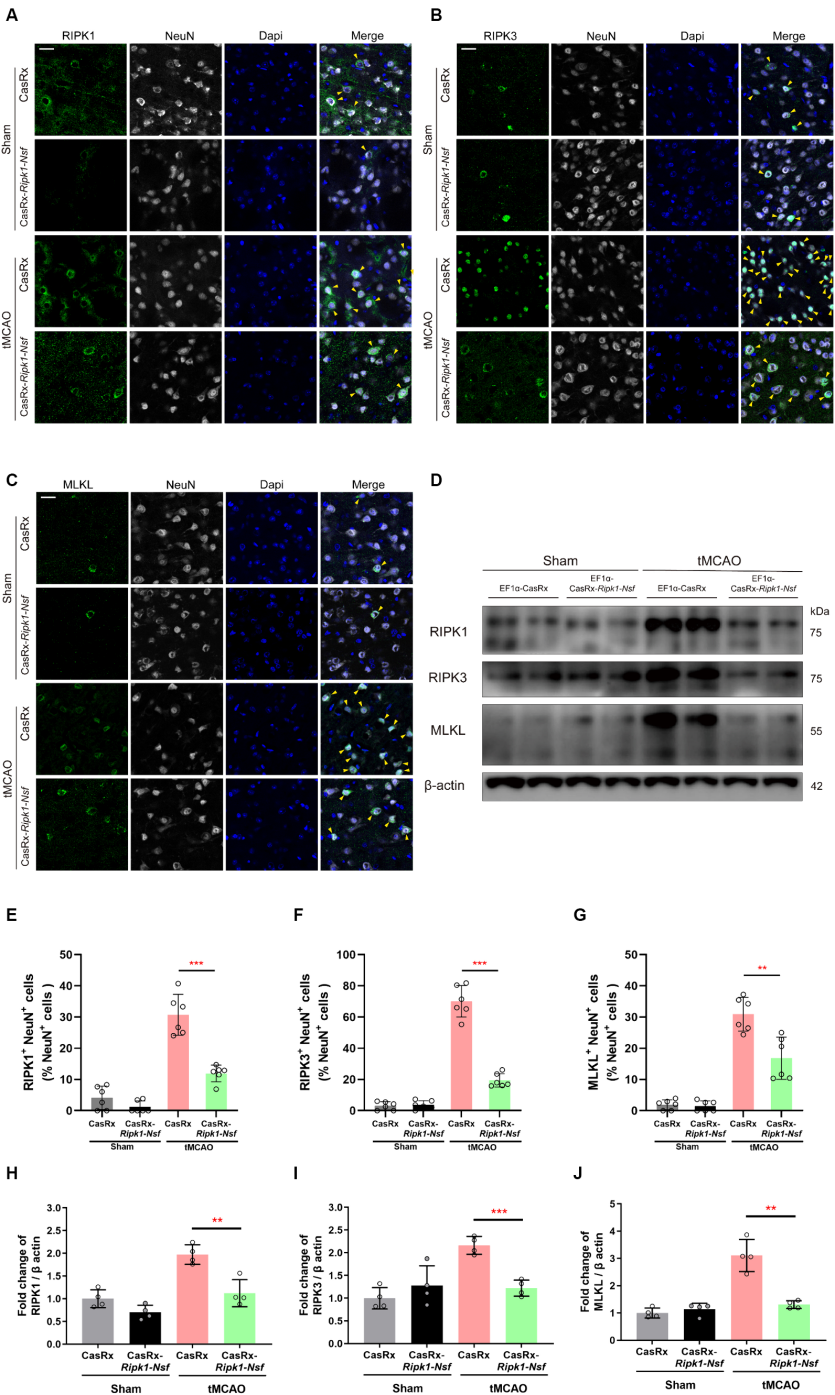
Effect of *Ripk1* and *Nsf* knockdown on post-stroke cerebral necroptosis mediated by RIPK1/RIPK3/MLKL signaling. **(A–C)** Representative confocal images illustrate the staining of RIPK1 (green) and NeuN (gray), as well as RIPK3 (green) and NeuN (gray), and MLKL (green) and NeuN (gray) in brain sections from the Sham and tMCAO (48 h) groups treated with CasRx-*Ripk1*-*Nsf* or CasRx. Yellow arrowheads indicate the colocalization of RIPK1 and NeuN, RIPK3 and NeuN, and MLKL and NeuN. Scale bar, 25 μm. **(D)** Expression levels of RIP1, RIP3, and MLKL in lateral cortex tissues of the peri-infarct area 48 h after sham-operation/tMCAO in sham group and tMCAO group. **(E–G)** Percentage of RIPK1^+^NeuN^+^ cells in NeuN^+^ cells, percentage of RIPK3^+^NeuN^+^ cells in NeuN^+^ cells, and percentage of RIPK3^+^NeuN^+^ cells in NeuN^+^ cells. *n* = 6 in four groups, data are presented as mean ± SEM, *p < 0.05, **p < 0.01, and ***p < 0.001, by unpaired *t* test. **(H–J)** Statistical analysis of immunoblotting revealed a decrease in the expression levels of RIPK1, RIPK3, and MLKL in the CasRx-*Ripk1*-*Nsf* group 48 h after tMCAO, compared with the vehicle AAV group. *n* = 4 in four groups, data are presented as mean ± SEM, *p < 0.05, **p < 0.01, and ***p < 0.001, by unpaired *t* test.

To elucidate the process by which NSF and RIPK1 sequentially initiate necroptosis in neurons, we performed immunofluorescence staining to determine the colocalization of RIPK1 and NeuN, RIPK3 and NeuN, and MLKL and NeuN in cerebral frozen sections. Additionally, Western blot analysis was performed to assess the relative expression levels of RIPK1, RIPK3, and MLKL in the ipsilateral brain peri-infarct tissue and sham-operated brain tissue. Mice treated with CasRx-*Ripk1*-*Nsf* exhibited a lower degree of colocalization between RIPK1 and NeuN in NeuN^+^ cells 48 h after tMCAO, compared with vehicle group (30.70% ± 6.57% vs. 11.93% ± 2.67%, *p* < 0.0001, *n* = 6, Figures 4A,E). Furthermore, the ratio of RIPK3^+^ NeuN^+^/NeuN^+^ cells and MLKL^+^ NeuN^+^/NeuN^+^ cells was decreased in mice treated with CasRx-*Ripk1*-*Nsf* compared with vehicle group, 48 h after tMCAO (ratio of RIPK3^+^ NeuN^+^/NeuN^+^ cells: 70.04% ± 10.08% vs. 19.43% ± 4.30%, *p* < 0.0001, *n* = 6, Figures 4B,F; ratio of MLKL^+^ NeuN^+^/NeuN^+^ cells: 30.91% ± 5.39% vs. 16.81% ± 6.75%, *p* = 0.003, *n* = 6, Figures 4C,G). Consistent with the immunofluorescence staining results, Western Blot analysis also demonstrated a decrease in the expression levels of RIPK1, RIPK3, and MLKL in mice treated with CasRx-*Ripk1*-*Nsf* compared with vehicle group, 48 h after tMCAO (RIPK1: 1.97 ± 0.21 vs. 0.12 ± 0.30, *p* = 0.004, *n* = 4; RIPK3: 2.16 ± 0.19 vs. 1.22 ± 0.18, *p* = 0.004, *n* = 4; MLKL: 3.11 ± 0.59 vs. 1.31 ± 0.14, *p* < 0.001, *n* = 4, Figures 4D,H–J). Additionally, in the two sham-operated group, there were no statistically significant differences in the colocalization of RIPK1 and NeuN or the expression levels of RIPK1 in mice treated with CasRx-*Ripk1*-*Nsf*, compared with vehicle group (ratio of RIPK1^+^ NeuN^+^/NeuN^+^ cells: 4.12% ± 3.75% vs. 1.22% ± 1.19%, *p* = 0.12, *n* = 6; 1.00 ± 0.20 vs. 0.70 ± 0.16, *p* = 0.054, *n* = 4). This suggests that under physiological conditions, RIPK1 is barely expressed. The results of both immunofluorescence staining and Western blot analysis indicate that knockdown of *Ripk1* and *Nsf* mediated by CasRx ameliorates necroptosis in neurons through the RIPK1/RIPK3/MLKL signaling pathway.

## Discussion

**Figure 5:**
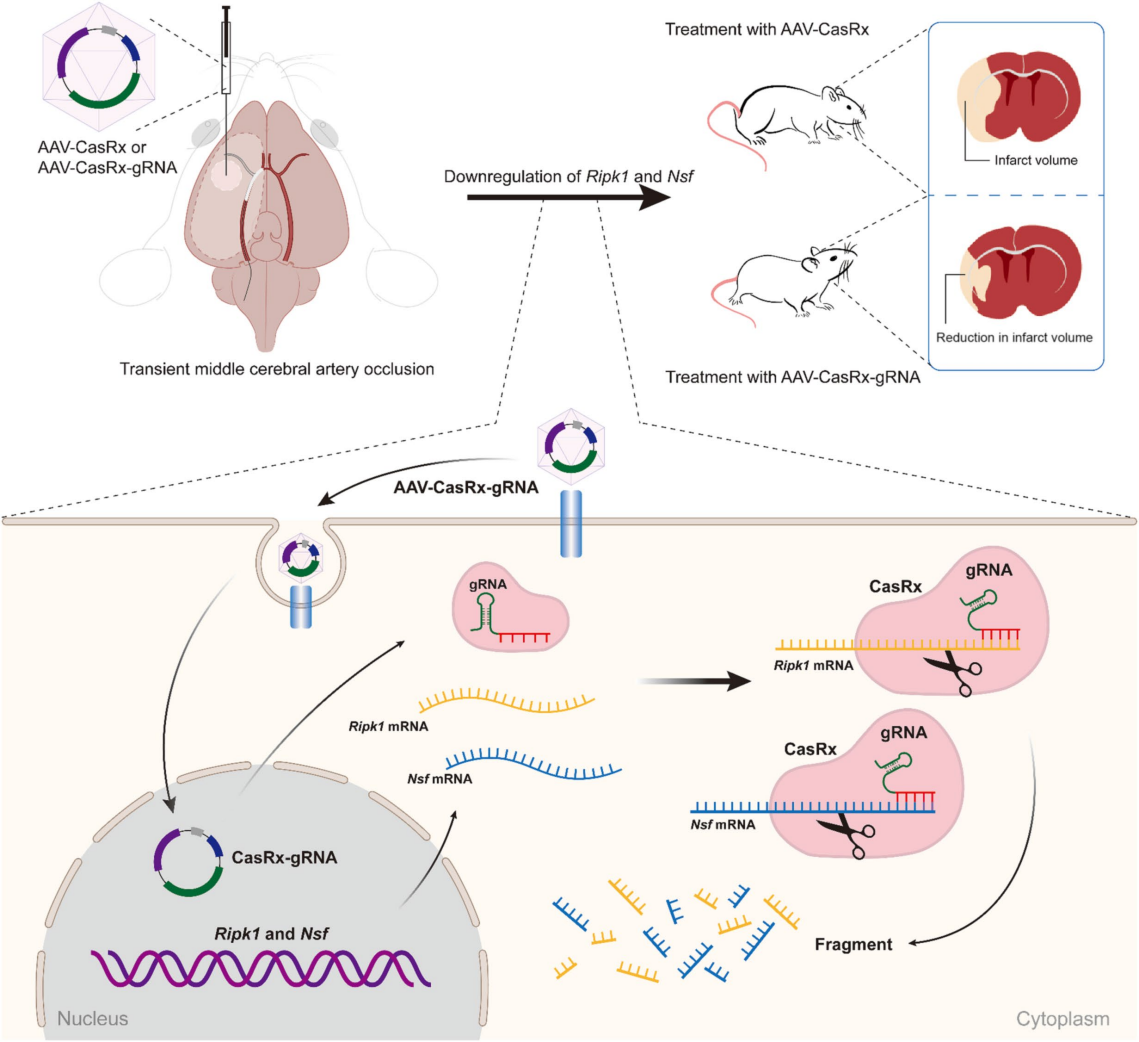
Schematic illustrating the effect of *Ripk1* and *Nsf* knockdown on ischemic stroke. The RNA-targeting CRISPR system, CasRx, downregulates *Ripk1* mRNAs and *Nsf* mRNAs to relieve the necroptosis triggered by ischemic stroke, thereby ameliorating stroke volume and neurological deficits in mice.

In this study, we initially screened the gRNAs with the highest knockdown efficiency for the *Ripk1* and *Nsf in vitro*. These gRNAs were then incorporated into the CasRx-*Ripk1*-*Nsf* plasmid, which was subsequently packaged into AAV9. Through the administration of AAV-EF1α-CasRx-*Ripk1*-*Nsf* into the striatum and S1BF regions of murine brains, we demonstrated that the knockdown of *Ripk1* and *Nsf* led to a reduction in both infarct and edema volume, accompanied by an improvement in neurological deficits in a mouse model of tMCAO. Importantly, the simultaneous knockdown of *Ripk1* and *Nsf* exerted neuroprotective effects on ischemic stroke by modulating the RIPK1/RIPK3/MLKL signaling pathway associated with necroptosis in neurons. These findings highlight the potential of *Ripk1* and *Nsf* knockdown as a neuroprotective strategy for mitigating cerebral damage and ameliorating neurological deficits following ischemic stroke (see Figure 5).

Necroptosis, a special manner of regulated necrotic cell death distinct from cell apoptosis or death (Degterev et al., 2005), is initiated by the activation of certain tumor necrosis factor receptor (TNFR) family members, including TNFR1 (Micheau and Tschopp, 2003), FAS (Strasser et al., 2009), tumor necrosis factor-related apoptosis-inducing ligand receptor 1 (TRAILR1), and TRAILR2 (Jouan-Lanhouet et al., 2012). Upon stimulation by their respective cognate ligands (TNF, FASL, and TRAIL), these receptors recruit RIPK1 through homotypic binding of their death domain with that of RIPK1 (Degterev et al., 2005). Recently studies have identified ASIC1a as a new death receptor that recruits RIPK1 to its C-terminus, leading to the phosphorylation of RIPK1 and subsequent neuronal death under the acidosis induced by ischemic stroke (Wang et al., 2015). Additionally, it has been proposed that NSF, a structural chaperone of proteins, serves as a trigger that disrupts the auto-inhibition of ASIC1a, resulting in the recruitment of RIPK1 and the induction of neuronal death (Wang et al., 2020). Although there is no direct interaction between NSF and RIPK1, ASIC1a serves as the intermediary connecting them. In an acidotic environment, ASIC1a sequentially recruits NSF and RIPK1 to induce neuronal death. Thus, we expect to inhibit neuronal death by targeted knockdown of ASIC1a, NSF or RIPK1 at the beginning. As a transmembrane protein, ASIC1a plays pivotal roles in cell membrane stability and synaptic plasticity (Krishtal, 2003), so we refrained from knocking it down. Furthermore, the ability to modify multiple-gene simultaneously would help to elucidate and control the gene interactions and networks underlying complex cellular functions. Among recent studies, strategies for simultaneously regulating multiple genes are emerging continuously. For instance, the downregulation of multiple oncogenes through engineered CasRx has demonstrated a stronger anticancer effect on bladder cancer (Zhan et al., 2023). And CRISPR-engineered T cells characterized by multi-gene regulation improved antitumor immunity in patients with refractory cancer (Stadtmauer et al., 2020). Thus, considering the synergistic effects of NSF and RIPK1 in inducing neuronal death, we chose to simultaneously downregulate NSF and RIPK1 in the hopes of a stronger neuroprotective effect than downregulating either alone. And the results in this study demonstrated reduced stroke volume and improved neuronal deficits after stroke intervened by CasRx-*Ripk1*-*Nsf*, which suggested that advances in CRISPR/CasRx-mediated multi-gene therapy are paving the way for future therapeutic applications in ischemic stroke.

The RIPK1/RIPK3/MLKL signaling pathway, following the initiation of NSF and the recruitment of RIPK1, complements to the mechanism underlying neuronal death induced by the conformational signaling of ASIC1a. In addition to its role as a critical regulator of necroptosis, RIPK1 also promotes a series of parallel detrimental signaling cascades that can induce varying degrees of neuroinflammation in neurodegenerative diseases (Zhang et al., 2013; Ransohoff, 2016; Chen et al., 2018), facilitated through various forms of ubiquitylation, deubiquitylation, and phosphorylation. Moreover, the involvement of NSF in ischemic stroke remains controversial. Peptides that inhibit NSF activity and block exocytosis have shown promise in exerting an antithrombotic effect on ischemic stroke (Matsushita et al., 2005). Protein–protein coupling/uncoupling enables NSF to exert either its active or negative effect on AMPA receptor-mediated excitotoxicity in ischemic stroke (Ralph et al. 2001; Zou et al. 2005). Plausibly, there exists a mutually reinforcing relationship between NSF depletion and ischemic brain injury (Liu and Hu 2004; Yuan et al. 2018a, 2018b, 2021). While a protective effect of short hairpin RNA (shRNA) knockdown of *Nsf* on neuronal death induced by acidosis *in vitro* has been reported (Wang et al. 2020), further studies are required to elucidate the effect of *Nsf* knockout or knockdown alone on ischemic stroke or neuroinflammation *in vivo*.

To enhance the translational value of our findings, we use a mouse model of ischemia/reperfusion brain injury in this study, which closely resembles the situation of a patient undergoing recanalization after stroke onset. In rodent models with middle cerebral artery occlusion, the striatum is a major site of lesion (Tamura et al. 1981). Additionally, the S1BF region is involved in sensory function and is responsible for processing stimuli and planning subsequent behavioral responses (Bale et al. 2021). Therefore, our observation of reduced infarct volume in both the cortex and striatum suggests that S1BF and striatum are promising therapeutic targets. The improvement in sensorimotor function observed through behavior tests also indicates a regional correlates with the sites of virus injection. These findings underscore the importance of advancing our understanding of the specific functions of different brain regions, as it will facilitate the development of more precise and effective treatments for brain injuries.

There are various strategies to achieve downregulation or knockout of a desired gene. However, it is crucial to consider the potential impact on DNA alterations and the toxicity of unintended products. For instance, shRNA technologies have the ability to cleave or suppress desired transcripts but also have exhibit significant off-target effects (Jackson et al. 2003; Birmingham et al. 2006). The CRISPR-Cas9 editing system, a well-established DNA editing technology, carries the risk of permanent DNA alterations, such as complex deletions, insertions, and large rearrangements, which can occur unintentionally (Shin et al. 2017; Kosicki et al. 2018). Notably, specific inhibitors of RIPK1 have been developed since the identification of necroptosis (Degterev et al. 2005). Although antagonist of RIPK1 and its analogues/derivatives provide new prospects for the prevention and treatment of multiple diseases (Mohammed et al. 2021; Yuk et al. 2021; Zeng et al. 2022), their clinical application is limited by metabolic instability (Geng et al. 2017) and deficiencies in off-target effects (Takahashi et al. 2012; Degterev et al. 2013). As an emerging gene editing technology, Cas13-mediated knockdown demonstrates comparable or higher efficiency than RNAi knockdown, with substantially reduced off-target effects (Abudayyeh et al. 2017; Cox et al. 2017; Konermann et al. 2018). Among the Cas13 family of proteins, CasRx has the smallest size, allowing for AAV packaging when paired with a CRISPR array (encoding multiple guide RNAs). Moreover, the risks associated with permanent DNA alterations in standard CRISPR-Cas9 editing can be avoided by the using RNA-targeting CRISPR system CasRx. While previous reports claimed that the CRISPR-Cas13a gene-editing system induced collateral cleavage of RNA in glioma cells (Wang et al. 2019), it is still important to consider whether CasRx also induces collateral cleavage in therapeutic applications due to its short-term effect of targeting cleavage. The efficiency and specificity of RNA editing by CasRx *in vivo* make it a potentially useful tool for treating other diseases that require downregulation of specific gene products.

It is worth noting that the current delivery method using AAV injection has certain drawbacks, including prolonged expression time of 7 to 14 days and the inevitably invasive manipulation. However, a recent study introduced a novel delivery tool using engineered extracellular vesicles (EVs), which can be loaded with CRISPR-CasRx and gRNA complexes to rapidly and transiently perturb the expression of target genes (Li et al. 2023). This emerging delivery method effectively addresses the issue of prolonged expression time associated with AAV delivery. If it becomes feasible to achieve targeted delivery of EVs loaded with CRISPR-CasRx and gRNA complexes to injured brain tissue during intra-arterial thrombectomy for ischemic stroke, it would overcome the limitation of invasiveness and present an intriguing challenge for future research.

## Conclusion

In summary, we demonstrated that localized knockdown of *Ripk1* mRNA and *Nsf* mRNA mediated by CRISPR-CasRx in the striatum and S1BF leads to a reduction in stroke size and an improvement in neurological deficits in a mouse model of tMCAO. These effects are mediated through the regulation of the RIPK1/RIPK3/MLKL signaling pathway involved in neuronal necroptosis. Thus, CasRx-mediated knockdown of *Ripk1* and *Nsf* holds promise as a neuroprotective strategy and provides a foundation for future gene therapy-based treatments for ischemic stroke.

## Data availability statement

The raw data supporting the conclusions of this article will be made available by the authors, without undue reservation.

## Ethics statement

The animal study was approved by Beijing China-Japan Friendship Hospital Ethics Committee. The study was conducted in accordance with the local legislation and institutional requirements.

## Author contributions

XS: Conceptualization, Data curation, Formal Analysis, Funding acquisition, Investigation, Methodology, Project administration, Resources, Software, Supervision, Validation, Visualization, Writing – original draft, Writing – review & editing. YL: Conceptualization, Data curation, Investigation, Methodology, Writing – original draft, Writing – review & editing. SL: Data curation, Supervision, Validation, Writing – review & editing. YW: Investigation, Project administration, Writing – review & editing. LC: Supervision, Validation, Writing – review & editing. TL: Supervision, Validation, Visualization, Writing – review & editing. FL: Resources, Validation, Visualization, Writing – review & editing. DP: Conceptualization, Data curation, Formal Analysis, Funding acquisition, Investigation, Methodology, Project administration, Resources, Software, Supervision, Validation, Visualization, Writing – review & editing.
