# Supplementary material for: Downregulation of Ripk1 and Nsf mediated by CRISPR-CasRx ameliorates stroke volume and neurological deficits after ischemia stroke in mice
Source: Front Aging Neurosci. 2024 Jun 11;16:1401038. doi: 10.3389/fnagi.2024.1401038 (PMC11197154; doi:10.3389/fnagi.2024.1401038)
Supplement: Supplementary file 1 [file Data_Sheet_1.PDF]

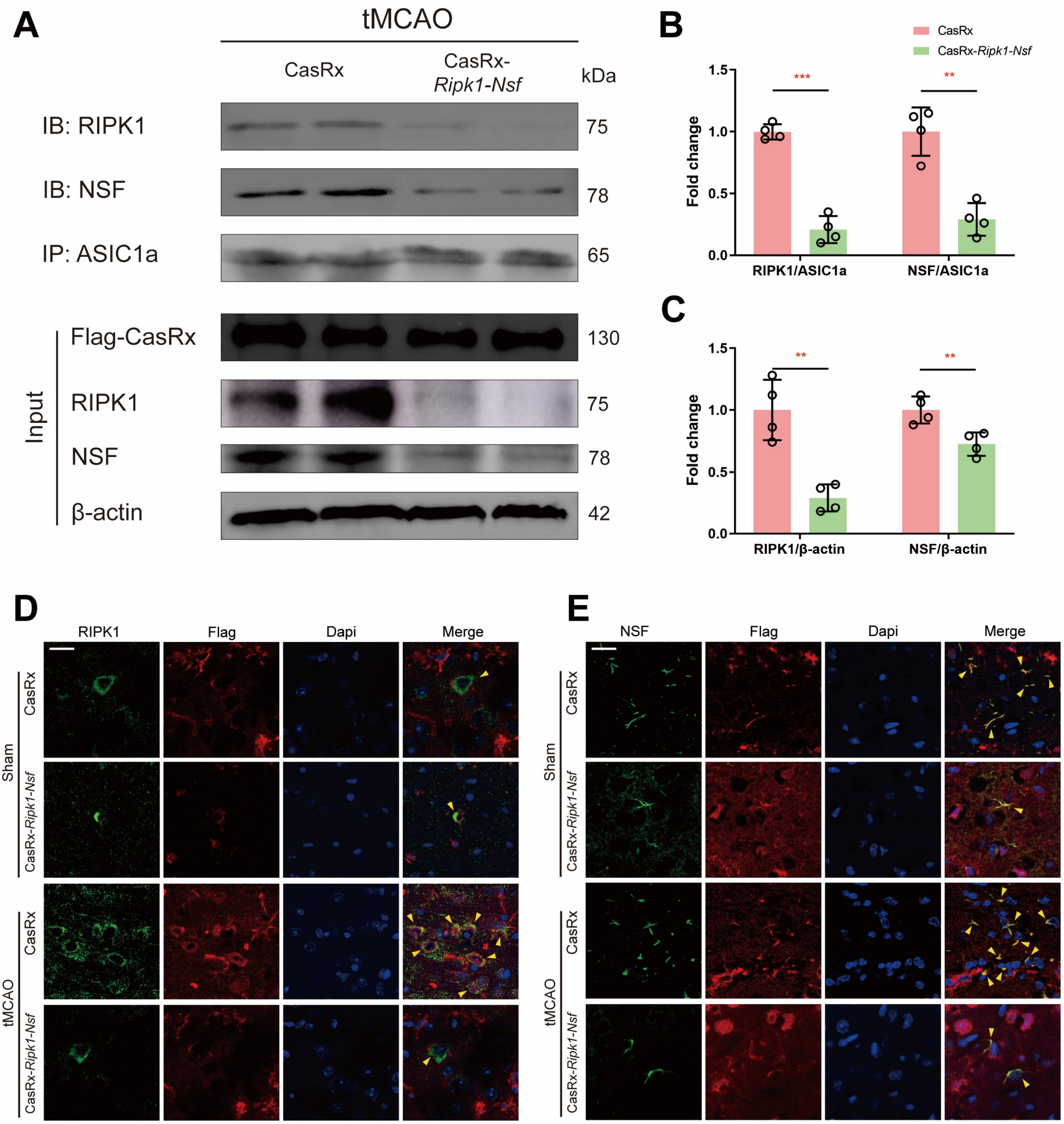
A
tMCAO
CasRx
CasRx-
Ripk1-Nsf
kDa
IB: RIPK1
75
IB: NSF
78
IP: ASIC1a
65
Input
Flag-CasRx
130
RIPK1
75
NSF
78
β-actin
42
B
CasRx
CasRx-Ripk1-Nsf
Fold change
***
**
RIPK1/ASIC1a
NSF/ASIC1a
C
Fold change
**
**
RIPK1/β-actin
NSF/β-actin
D
RIPK1
Flag
Dapi
Merge
Sham
tMCAO
CasRx
CasRx-Ripk1-Nsf
E
NSF
Flag
Dapi
Merge

Figure S1. Effect of *Ripk1* and *Nsf* knockdown after tMCAO *in vivo*.

(A) The expression levels of RIPK1 and NSF, which were pulled down from ASIC1a using co-immunoprecipitation, were evaluated in the CasRx-*Ripk1*-*Nsf* group compared with CasRx control group 2 hours after tMCAO/sham. CasRx was labeled with a Flag-tag.

(B and C) The summary data indicated a decreased association between RIPK1 and ASIC1a, as well as between NSF and ASIC1a, after tMCAO in the CasRx-*Ripk1*-*Nsf* group compared with the CasRx control group. Additionally, the total levels of RIPK1 and NSF also decreased after tMCAO. n = 4, data are presented as mean ± SEM, *$P < 0.05$, **$P < 0.01$, ***$P < 0.001$, by unpaired t test.

(D and E) Representative confocal images illustrate the colocalization of RIPK1 (green) and Flag (red), as well as NSF (green) and Flag (red), in brain sections from the Sham and tMCAO (2 hours) groups treated with CasRx-*Ripk1*-*Nsf* or CasRx. Yellow arrowheads indicate the colocalization. Scale bar, 20 μm. n = 4 for the determination of RIPK1 while n = 3 for the determination of NSF.

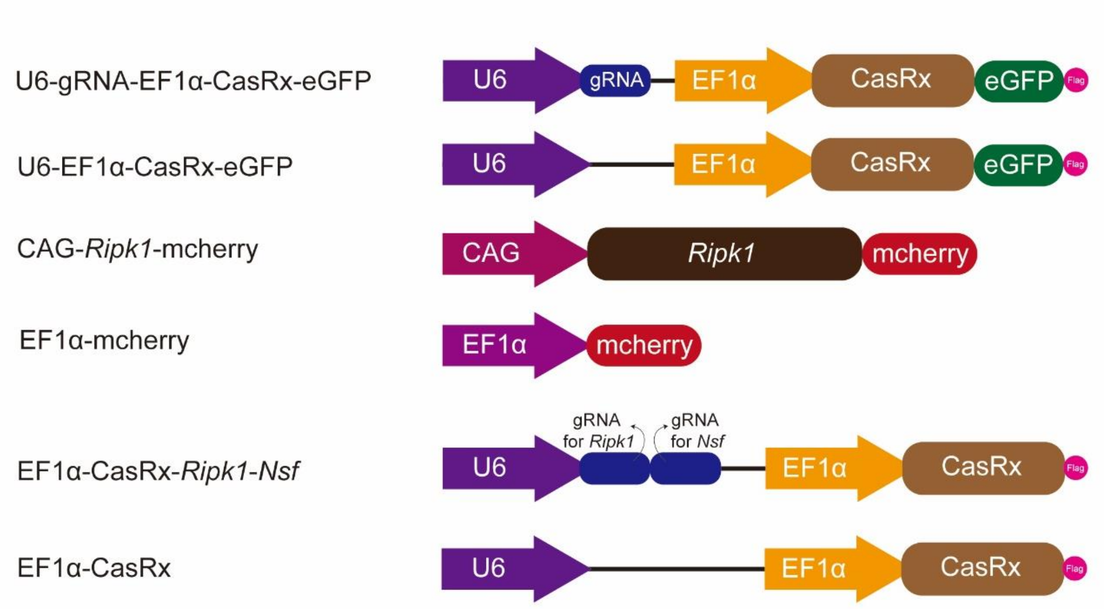

Figure S2. A schematic of plasmids mainly used in this study

A schematic of plasmids mainly used in this study. CasRx was labeled with a Flag-tag.

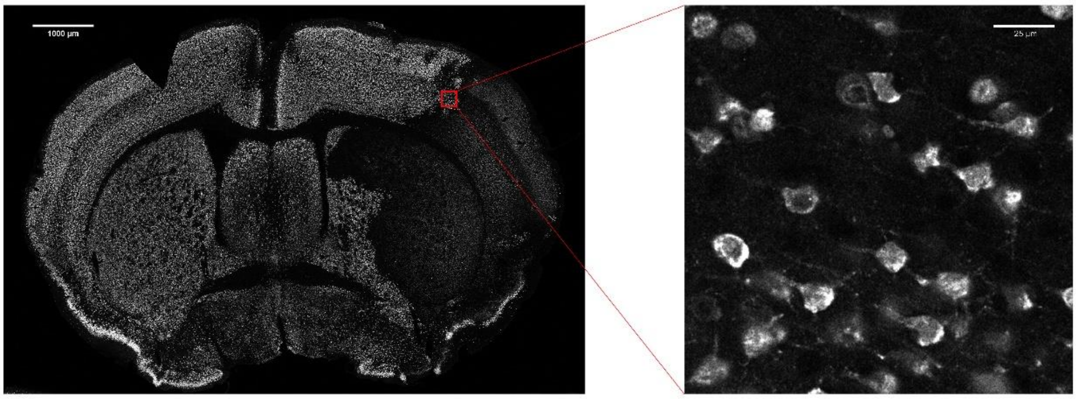

Figure S3. A whole-brain image illustrating the representative location of the images captured by confocal laser scanning microscope.

A whole-brain image at 4x magnification with staining of NeuN to illustrate the representative location of the images captured in brain sections from the Sham and tMCAO (48 hours) groups treated with CasRx-*Ripk1*-*Nsf* or CasRx. Scale bar, 1000 µm (left) and 25 µm (right).

Supplementary-table 1. The Base Sequence of gRNAs for *Ripk1* and *Nsf*

Oligonucleotides	Base Sequence
gRNA 1 for *Ripk1*	5'-caggtcactggatgccatcttaatattgtcc-3'
gRNA 2 for *Ripk1*	5'-cttctgtggtaacacaaggacaccttcccgaa-3'
gRNA 3 for *Ripk1*	5'-atgacaaatccatggcttctgtggtaacac-3'
gRNA 4 for *Ripk1*	5'-tctgtgcatcatcttcccctcttccaagag-3'
gRNA 5 for *Ripk1*	5'-ttgcccttctccatgtactccatcaccagcg-3'
gRNA 6 for *Ripk1*	5'-tccttgtgtatcacacctttgtcatgt-3'
gRNA 7 for *Ripk1*	5'-atcggctatcttaatgtgaaagtcacgatc-3'
gRNA 8 for *Ripk1*	5'-atgtcattcaggtgttcgggtgccatgtagt-3'
gRNA 9 for *Ripk1*	5'-cctgttcccagattttatgcagatcacg-3'
gRNA 10 for *Ripk1*	5'-aggctgatgatctcccttggacagtactc-3'
gRNA 11 for *Ripk1*	5'-aactgtgcagcgatccaggttgttctg-3'
gRNA 12 for *Ripk1*	5'-caattccacgactcgttgtgctgggatcag-3'
gRNA 13 for *Ripk1*	5'-tggttgtgaattcagtccaacatccatat-3'
gRNA 14 for *Ripk1*	5'-tgttcatcagtcagactagtggtgttatca-3'
gRNA 1 for *Nsf*	5'-tcctcaccatcacatgctggccagactgg-3'
gRNA 2 for *Nsf*	5'-gaaccactgacggatgggtcctcagcgt-3'
gRNA 3 for *Nsf*	5'-aatacaaggcaacttctatgtcctgtccaatag-3'
gRNA 4 for *Nsf*	5'-gttggagtcgatgttcttcttctgcagg-3'
gRNA 5 for *Nsf 1*	5'-atgaactcggcggccatcttgtcggtgtcgt-3'
gRNA 6 for *Nsf*	5'-agaaggcctggttgttgaactgctggatg-3'
gRNA 7 for *Nsf*	5'-ggatccatggcttcaatgtccttcaccagt-3'
gRNA 8 for *Nsf*	5'-cattcagcatcttgccaatctgtcgagcc-3'
gRNA 9 for *Nsf*	5'-aatgttagcctctgattctcccacatacttgtt-3'
gRNA 10 for *Nsf*	5'-atgatgatgtgcaagccactgttagcacc-3'
gRNA 11 for *Nsf*	5'-tcttccgggtcgaaggagagcttcgtctatc-3'
gRNA 12 for *Nsf*	5'-aaggatctggagtcgacccttctcatctggc-3'
gRNA 13 for *Nsf*	5'-ccagctccttgatgtccacatctgcagacagt-3'
gRNA 14 for *Nsf*	5'-accaatggggacataatcgagcagcctctc-3'
gRNA 15 for *Nsf*	5'-caatgttgggcacgtggatggtggtgctg-3'

Supplementary-table 2. The qPCR Primers of *Ripk1* and *Nsf*

Oligonucleotides	Base Sequence
Ripk1 qPCR primers Forward	5'-gtgatacacaaggacctgaa-3'
Ripk1 qPCR primers Reverse	5'-cttagtggtgctgctcactt-3'
Nsf qPCR primers Forward	5'-acaagatggccgccgagttc-3'
Nsf qPCR primers Reverse	5'-ccttcaggatgctgggatcc-3'
